# Circular RNA hsa_circ_0000751 serves as a microRNA-488 sponge to suppress gastric cancer progression via ubiquinol-cytochrome c reductase core protein 2 regulation

**DOI:** 10.1080/21655979.2021.1983974

**Published:** 2021-10-27

**Authors:** Danwen Wang, Fei Su, Maohui Feng

**Affiliations:** a Department of Gastrointestinal Surgery, Zhongnan Hospital of Wuhan University, Wuhan, Hubei, China; b Clinical Medical Research Center of Peritoneal Cancer of Wuhan, Wuhan, Hubei, China; c Clinical Cancer Study Center of Hubei Provence, Wuhan, Hubei, China; d Hubei Key Laboratory of Tumor Biological Behaviors, Wuhan, Hubei, China; e Department of Oncology, The First Hospital of Lanzhou University, Lanzhou, Gansu, P.R. China

**Keywords:** Circular RNA, hsa_circ_0000751, miR-488, UQCRC2, gastric cancer

## Abstract

Circular RNAs (circRNAs) are RNA molecules that do not encode proteins but are known to regulate tumor progression. This study was designed to explore the underlying mechanism driving circRNA-mediated modulation of gastric cancer (GC). Bioinformatics analysis of gene chip GSE83521 was used to identify multiple circRNAs that were differentially regulated in matched GC and adjacent normal tissues. The circRNA with the largest variation in expression (hsa_circ_0000751) was selected for further examination. The expression profile of hsa_circ_0000751 and its target-specific interactions with microRNAs (miRNAs) and downstream gene transcripts were determined using quantitative real-time polymerase chain reaction, luciferase reporter assays, and rescue assays in human tissues and cells. The relationship between hsa_circ_0000751 expression and the clinicopathological parameters of 25 GC patients was analyzed. Furthermore, ubiquinol-cytochrome c reductase core protein 2 (UQCRC2), a GC suppressor, was detected via western blot analysis. The results showed that hsa_circ_0000751 levels were markedly downregulated in GC tissues and cell lines, which were also inversely proportional to the stage of tumor-node-metastasis (TNM) classification, tumor volume, and lymph node metastasis in GC patients. Conversely, hsa_circ_0000751 overexpression suppressed tumor progression, migration, and invasion *in vitro* and *in vivo*. From our results, we showed that hsa_circ_0000751 may serve as a miRNA sponge to suppress the activity of miR-488, thereby increasing the expression of the miR-488-target gene, UQCRC2, and limiting GC progression. Given its negative regulation of oncogenic miRNAs, the hsa_circ_0000751/miR-488/UQCRC2 axis may be crucial in the development of novel GC therapies.

## Introduction

Gastric cancer (GC) ranks fifth among all cancers in terms of occurrence and is the fourth leading cause of cancer-related deaths. In 2020, approximately 1,089,000 newly diagnosed cases and a whopping 769,000 mortalities were noted worldwide [1]. Although multiple diagnostic and therapeutic advancements have occurred, the total five-year survival rate among all diagnosed cases is only 30% [2]. Multiple factors, including genetic abnormalities, epigenetic changes, and abnormal signaling, are known to regulate the development, progression, and metastasis of GC [3]. Therefore, it is of utmost urgency to determine alternative molecular and genetic targets of GC and identify novel biomarkers for early diagnosis and treatment.

Circular RNAs (circRNAs) were initially observed, using electron microscopy, in the Sendai virus in 1976 [4]. CircRNAs, particularly when compared to the classical linear-shaped mRNA or non-coding RNA, are unique in their circular formation, bound by covalent bonds, and carry a back splice region between the 5ʹ- and 3ʹ- ends [5,6]. They are also reported to be highly stable, evolutionarily conserved, and ubiquitously available in specific tissues [7]. Recent evidence points toward the function of circRNAs in cancers; in particular, circRNAs act as microRNA (miRNA) sponges to regulate tumorigenesis through their mi-RNA binding sites [8]. One such example is the circNRIP1-mediated promotion of GC progression by sponging microRNA-149-5p [9]. In another example, circPSMC3 was shown to suppress GC cell proliferation and invasion via the circPSMC3/miR-296-5p/PTEN axis [10] So there are diverse functions of circRNAs in the development of GC.

According to current consensus, miRNAs are a highly conserved network of small regulatory ncRNAs that are known to modulate multiple biological functions [8]. Previous studies have shown that miR-488 is overexpressed in primary osteosarcoma tissues, promoting the proliferation of osteosarcoma cells [11]. Furthermore, miRNA-488 acts as a tumor suppressor in non-small cell lung cancer (NSCLC) by inhibiting cell migration and invasion by targeting eukaryotic translation initiation factor 3a [12]. However, the understanding of the involvement of miRNA-488 in GC remains limited and warrants further research.

Ubiquinol-cytochrome c reductase core protein 2 (UQCRC2) is a pivotal mitochondrial respiratory complex III subunit that plays an important role in the mitochondrial oxidative respiratory chain [13]. Recent evidence points toward a close relationship between UQCRC2 and human diseases, particularly cancers. Our previous study revealed a weak presence of UQCRC2 in GC tissues, as evidenced by quantitative real-time polymerase chain reaction (qRT-PCR). We further confirmed the feasibility of using UQCRC2 as a stand-alone diagnostic biomarker for GC disease survival, wherein we found that the expression level of UQCRC2 showed a significant negative correlation with disease progression [14].

Here, we aimed to explore the roles and potential mechanisms of hsa_circ_0000751 in the development of GC. Our research demonstrated a functional loop between hsa_circ_0000751, miR-488, and UQCRC2. In brief, we demonstrated that hsa_circ_0000751 is markedly suppressed in both GC tissue samples and cells, which positively affected the clinical stage, tumor volume, and lymph node metastasis. We also demonstrated that hsa_circ_0000751 inhibits GC progression by sponging miR-488, thereby regulating the expression of the tumor suppressor gene UQCRC2. Taken together, hsa_circ_0000751 has the potential to be an independent diagnostic marker and a likely target for GC therapy.

## Materials and methods

### Tissue samples

For the study, 25 samples of GC tumors and paracancerous tissues were retrieved from patients who underwent surgery at the Zhongnan Hospital (Wuhan, China) between December 2016 and December 2019. After collection, all tissue samples were immediately stored at −80°C. All patients selected for sample collection were screened according to a postoperative pathological diagnosis. None of these patients had received radiotherapy, chemotherapy, or any other form of treatment prior to surgery. The clinicopathological factors are summarized in Table 1. Before inclusion in the study, the patients provided written informed consent. This study abided by the recommendations of the Declaration of Helsinki and was approved by the Medical Ethics Committee of Wuhan University (approval number: 2,015,011).

**Table 1.** Relationship between hsa_circ_0000751 expression and clinicopathological factors

Characteristics	Group	Low expression (n = 14)	High expression (n = 11)	P value
Gender	Male	8	5	0.569
	Female	6	6	
Age(year)	≥50	9	7	0.214
	<50	5	4	
Lymph node metastasis	Yes	10	2	0.010*
	No	4	9	
Tumor size(cm)	<4	5	8	0.043*
	≥4	9	3	
TNM stage	I–II	8	10	0.025*
	III–IV	6	1	

Statistical analyses were by Pearson’s χ^2^ test. *, P < 0.05 was considered significant.

### Cell lines and culture

Human GC cell lines (MKN-28, AGS, MKN-45, BGC-823, MGC-803, and HGC-27) and normal human gastric epithelial cells-1 (GES-1) were obtained from the American Type Culture Collection. All cells were cultured in a 37°C and 5% CO_2_ humidified environment and in RPMI-1640 (Gibco, Thermo Fisher Scientific Inc., Waltham, MA, USA), 10% fetal bovine serum (FBS; Gibco, Thermo Fisher Scientific Inc.), and 1% penicillin–streptomycin [15].

### Plasmid construction and cell transfection

Full-length hsa_circ_0000751 cDNA was amplified in 293 T cells before cloning into the pLCDH-ciR vector (Geneseed, Guangzhou, China) with a front and back circular frame. The negative control was constructed without the addition of the hsa_circ_0000751 sequence. The following plasmids were purchased from Genechem (Shanghai, China): UQCRC2, UQCRC2 siRNA, hsa_circ_0000751 siRNA, miR-488 mimic and inhibitor, and two scrambled negative control miRNAs (specifically, mimic-NC for miR-488 mimics and inhibitor-NC for miR-488 inhibitors). The siRNA sequences used were as follows: si-UQCRC2: 5ʹ-ATCCTCGACGCGATGAGA-3ʹ and si-hsa_circ_0000751: 5ʹ-CCGCAGGCTCCCAGTCCCAAT-3ʹ. Lipofectamine 3000 (Thermo Fisher Scientific, Waltham, MA, USA) was used for plasmid incorporation into cells, following the manufacturer’s guidelines. Finally, total RNA and protein were harvested 48 h post-transfection, following standard protocols [16].

### Microarray analysis

The circRNA expression profile (source: GSE83521) was downloaded from the National Center for Biotechnology Information Gene Expression Omnibus (GEO). GSE83521 was compiled using data from six GC tissues and six matched non-tumor tissue samples, based on GPL19978 (Agilent-069978 Arraystar Human CircRNA microarray V1). RNA sequencing and microarray data were preprocessed using R software (version 3.5.0, available online: https://www.r-project.org/) and packages. Differentially expressed circRNAs were identified based on the following criteria: fold alterations >2 and *p* values < 0.05.

### qRT-PCR

TRIzol (Takara, Shiga, Japan) was used to harvest total RNA from cultured cells and human tissues, and the NanoDrop was used for quantification of the total extracted RNA. Next, 10 μg of the total extracted RNA was treated with 40 U of RNase R (Epicenter Technologies, Madison, USA) by incubating at 37°C for 15 min. CircRNA and mRNA analyses were performed by reverse transcribing RNA into complementary DNA (cDNA) using a PrimeScript™ RT Master Mix reagent kit (Takara). Genomic DNA (gDNA) was isolated using the QIAamp DNA Mini Kit (QIAGEN, Germany). Transcript levels were assessed using qRT-PCR with SYBR Premix Ex Taq™ (Takara). Endogenous GAPDH and U6 were used to normalize the expression of the relevant transcripts. Finally, gene expression was quantified using the 2^−ΔΔCt^ method [17]. The unique primers used for the detection of circRNAs and miRNAs were purchased from Sangon Biotech (Shanghai, China) and are listed in Table 2.

**Table 2.** Sequences of primers used for quantitative RT-PCR assay

Primer ID	Primer sequences (5ʹ-3ʹ)
Circ-0000751 F	ATAACAACTGCTCAGAGTGCTA
Circ-0000751 R	CTCAGCTTCCTGTAGGATGTGC
miR-488 F	ACACTCCAGCTGCCTAGCAGCACAGAAATAT
miR-488 R	CTCAACTGGTGTGGTGGA
U6 F	CTCGCTTCGGCAGCACA
U6 R	AACGCTTCACGAATTTGCGT
UQCRC2 F	AATTTCGTCGTTGGGAAGTAGC
UQCRC2 R	ATGAGTCTGCGGATTCTGAAAG
GAPDH F	GGAGCGAGATCCCTCCAAAAT
GAPDH R	GGCTGTTGTCATACTTCTCATGG

### Western blot

Proteins were isolated from stably transfected cells at 90% confluence using the RIPA lysis buffer (Beyotime Biotechnology, Jiangsu, China). Their concentrations were quantified using a BCA protein assay kit (Beyotime Biotechnology, Jiangsu, China), and the proteins (30 µg/lane) were separated via SDS-PAGE before transfer to a polyvinylidene fluoride membrane (Millipore, Billerica, MA, USA). Following this, the membrane with the proteins was subjected to the following incubations and washes: 5% lipid-free milk solution at 37°C for 1.5 h, primary antibody at 4°C overnight (ON), HRP-conjugated secondary antibody (1:1,000; Abcam, Shanghai, China) for 2 h at 37°C, and three TBST buffer washes. Finally, the protein signals were visualized using an enhanced chemiluminescence detection system with a chemiluminescent HRP substrate (Millipore, MA, USA). The antibodies used for protein detection were as follows: anti-UQCRC2 (1:1000, Abcam, Shanghai, China), anti-GAPDH (1:5000; Cell Signaling Technology, MA, USA), anti-cleaved caspase-3 (1:200, Cell Signaling Technology, MA, USA), anti-Bcl-2 (1:1000, Abcam, Shanghai, China), anti-Bax (1:1000, Abcam, Shanghai, China), and anti-Bak (1:10,000, Abcam, Shanghai, China).

### Luciferase reporter assay

Dual fluorescein reporter gene analysis was performed using a Dual Luciferase Assay System Kit (Promega, Madison, WI, USA), following the manufacturer’s guidelines. Wild-type hsa_circ_0000751 3ʹ UTRs (WT hsa_circ_0000751 3ʹ UTRs), mutant hsa_circ_0000751 3ʹ UTRs (MUT hsa_circ_0000751 3ʹ UTRs), and UQCRC2 3ʹUTRs (MUT UQCRC2 3ʹUTRs) were amplified and cloned into the pmirGLO luciferase reporter vector (GeneCreat, Wuhan, China). Next, HEK-293 T cells were plated in 24-well plates in triplicate and co-incorporated with the corresponding plasmids and miR-488 mimics. Following a 48-h incubation, luciferase activity was assessed using the dual-luciferase reporter assay system (Promega). The data were normalized to the Renilla internal control.

### 5-ethynyl-20-deoxyuridine (EdU) assay

The proliferative capacity of GC was measured using the Cell-Light™ EdU DNA Cell Proliferation Kit (Ribobio, Guangzhou, China) and Cell Counting Kit (CCK)-8 (Bosterbio, Wuhan, China), according to the manufacturer’s guidelines [18]. To perform the EdU assay, MKN-45 and MGC-803 cells were exposed to 50 mM EdU for 2 h before fixation in 4% paraformaldehyde, and then stained with Apollo Dye Solution. Nuclear staining was done using Hoechst-33,342. Next, EdU-positive or proliferating cells were quantified using an Olympus FSX100 microscope (Olympus, Tokyo, Japan). Five randomly chosen fields per group were selected for the quantification of cell proliferation.

### CCK-8 assay

To perform the CCK-8 assay, gastric cells (5 × 10^3^ cells per well) were grown in 96-well plates for 24 h. Next, the cells were exposed to 10 μL CCK-8 solution (Dojindo, Tokyo, Japan) and incubated at 37°C for 90 min [19]. The absorbance of the cells was recorded at 450 nm using a microplate reader (Bio-Rad, Hercules, CA, USA).

### Colony-formation assay

The colony-forming capability of GC cells was evaluated using colony-formation assays [20]. In brief, GC cells were incorporated with the indicated plasmids and incubated for 24 h. Next, 500 cells from each cell line were grown in 6-well plates for 2 weeks without changing the medium. The formed colonies were then fixed with methanol for 20 min before staining with 0.1% crystal violet at room temperature, followed by photography and counting.

### Wound-healing assay and transwell invasion assay

Cells were allowed to grow to full confluence in 6-well plates at a density of 1 × 10^6^/well. Next, using a sterile pipette tip, the cell surface was scratched and the formation of a clear gap in the confluent cell layer was confirmed by observing the wells under a microscope. Following this, the old medium was removed, and new medium, with no serum, was added. Finally, the cells were imaged at 0 and 24 h post-injury to determine the width of the wound healing and compare it against the baseline.

For the transwell invasion assay, 1 × 10^5^ cells were plated in 500 μL of medium, with no serum, in the top Matrigel-coated (BD Biosciences, San Jose, USA) chamber of 24-well plates (Corning, NY, USA). Medium with 10% FBS was introduced into the bottom chamber as a chemoattractant. Following a 24 h incubation, cells that managed to migrate to the bottom surface were fixed in 4% paraformaldehyde, stained with 0.1% crystal violet, imaged, and quantified using at least five random fields of view (magnification: ×200, Olympus, Japan). All experiments were independently performed thrice.

### Animal experiments

Xenograft studies were performed in six-week–old BALB/c nude mice obtained from the Chinese Science Academy (Shanghai, China). For experimentation, 10 random mice were assigned to 2 groups of 5 mice each. Meanwhile, MKN-45 cells were stably incorporated with lentivirus-hsa_circ_0000751-overexpression vector or a negative control before injecting the transfected cells into the mice subcutaneously. In all cases, the forelimbs axilla received 5 × 10^6^ cells per mouse. Next, the tumor volume was monitored every week (volume = width^2^× length×1/2). All animal protocols were approved by the Committee on Animal Research of Wuhan University.

### Immunohistochemistry (IHC) analysis

Tumor tissue samples from mice were collected for IHC staining, as previously reported [21]. The excised tumors were fixed in 10% formalin, embedded in paraffin, and cut into 4 μm thick slices. Finally, the tissue slices were probed with primary anti-Ki-67 antibody (1:500, Abcam) before the collection of images under a microscope (Olympus, Japan) at an appropriate magnification. The positive staining rate indicated the proportion of positive GC cells.

### Terminal deoxynucleotidyl transferase dUTP nick end labeling (TUNEL assay)

Apoptosis was analyzed using a TUNEL assay. A TUNEL assay was used to calculate the level of fragmented DNA using the TUNEL apoptosis detection kit (Beyotime Institute of Biotechnology, Nantong, China) following the manufacturer’s guidelines [22]. Briefly, the cells were fixed with 4% paraformaldehyde for 30 min, washed with PBS, exposed to 0.3% Triton-X 100 in PBS for 5 min, equilibrated with 100 μL equilibration buffer at 37°C for 10 min, labeled with 50 μL TUNEL reaction buffer at 37°C for 1 h, followed by DAPI counterstaining for 1 min at 37°C in the dark, and finally mounted using an anti-fade mounting medium. The stained apoptotic cells were visualized using a fluorescence microscope (Carl Zeiss, Oberkochen, Germany).

### Statistical analysis

Data are presented as means ± standard deviations and analyzed with SPSS 20.0 (IBM, SPSS, Chicago, IL, USA) and GraphPad Prism version 7.0. Statistical significance (*p* < 0.05) was assessed using the Student’s *t*-test (comparing two different groups) and one-way analysis of variance (ANOVA) (comparing multiple groups). Correlations between hsa_circRNA_0000751 levels and clinical manifestations were assessed using the χ^2^ test. Pearson’s correlation coefficient analysis was employed to ascertain relationships between various factors.

## Results

### Identification and characterization of hsa_circ_0000751

**Figure 1.**
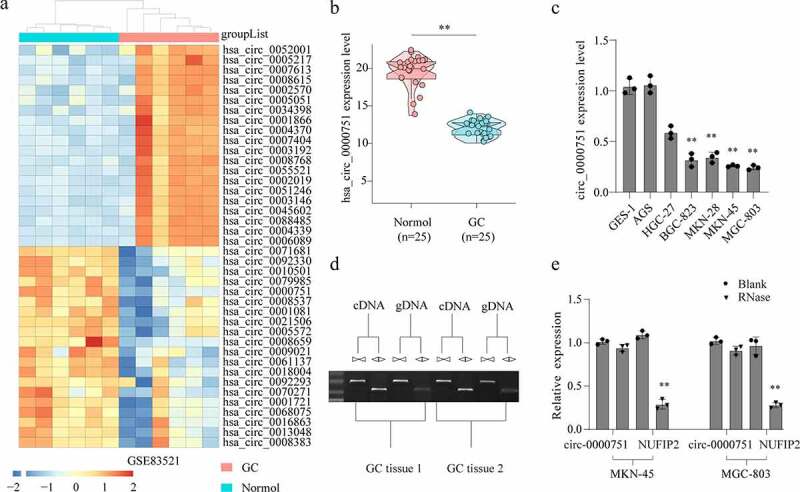
Identification and characterization of hsa_circ_0000751. (a) The heat map of circRNAs expression profiles in normal and GC tissues analyzed from GEO database (GSE83521). Red represents high level and blue indicates low level of transcript abundances. (b) Hsa_circ_0000751 expression was validated in GC tissues and adjacent normal tissues via qRT-PCR. (c) Hsa_circ_0000751 expression in GC cells lines and normal human gastric epithelial cell using qRT-PCR. (d) Gel electrophoresis shows that hsa_circ_0000751 can be amplified by divergent primers using total cDNA, but not genomic DNA (gDNA). (e) Relative expression of hsa_circ_0000751 and NUFIP2 mRNA in both MKN-45 and MGC-803 cells was detected by qRT-PCR in the presence or absence of RNase R. Data were represented as means ± S.D. of at least three independent experiments.**P < 0.01

To distinguish differentially regulated circRNAs in GC, we examined six matched GC and normal tissue samples from the GEO database (GSE83521). We identified 40 circRNAs with abnormal expression in GC tissues, carrying a fold change > 2 and p < 0.05, as shown in Figure 1(a). hsa_circ_0000751, also known as circNUFIP2 in the circBase (http://www.circbase.org/), showed the most significant downregulation in GC. To verify the above results, we also confirmed low hsa_circ_0000751 levels in 25 GC tissues and 25 adjacent tissues (Figure 1(b)) using qRT-PCR analysis. Moreover, we detected hsa_circ_0000751 expression in GES-1 and in multiple GC cells (AGS, SCG-7901, BGC-823, MKN-28, MKN-45, and MGC-803). According to our data, hsa_circ_0000751 had the lowest expression levels in MKN-45 and MGC-803 cells (Figure 1(c)). Therefore, these cell lines were selected for further studies.

Since hsa_circ_0000751 has been shown to originate from the mRNA splicing of NUFIP2 (nuclear FMR1 interacting protein 2), it was imperative to determine whether the hsa_circ_0000751 in our cells originated from a gene rearrangement event. Therefore, we constructed convergent and divergent primers to amplify the linear and circular RNAs, respectively, based on complementary DNA (cDNA) and genomic DNA (gDNA) from two randomly selected GC tissues. Using agarose gel electrophoresis, we demonstrated that hsa_circ_0000751 could be amplified from cDNA using divergent primers, but no amplification occurred from gDNA (Figure 1(d)). Furthermore, we utilized RNase R assay to reveal that hsa_circ_0000751, but not NUFIP2 mRNA, was resistant to RNase R (Figure 1(e)). Based on these data, it is evident that this RNA species is naturally circular.

### Effects of hsa_circ_0000751 on the malignant biological behavior of GC cells

**Figure 2.**
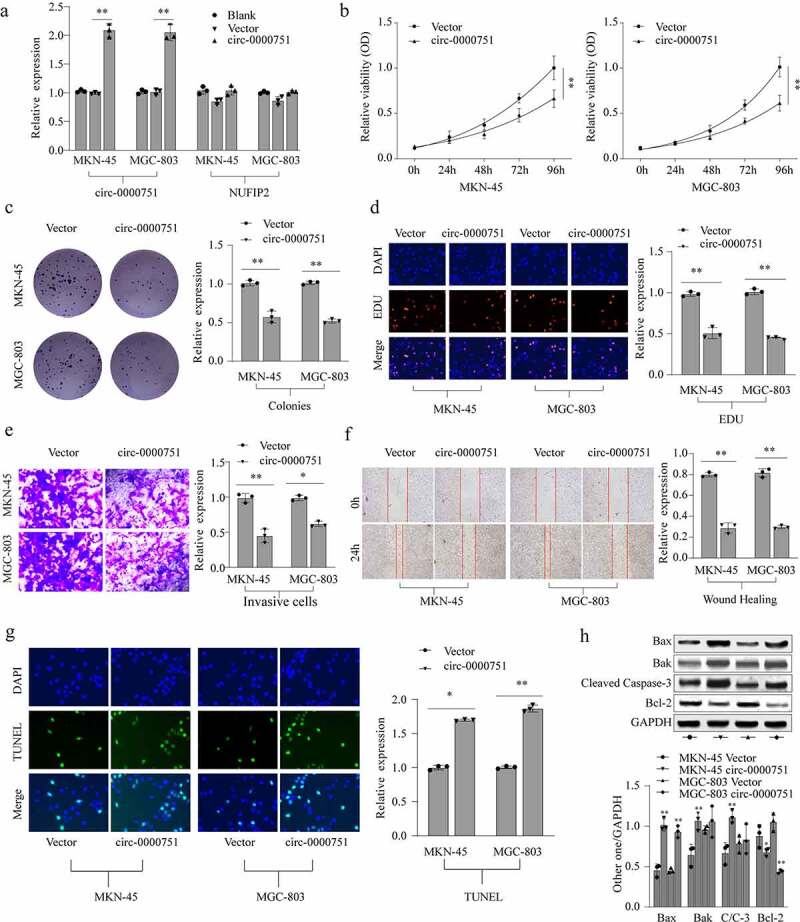
Hsa_circ_0000751 affects the proliferation, apoptosis and invasion abilities of GC cells. (a) The expression levels of hsa_circ_0000751 and NUFIP2 mRNA in MKN-45 and MGC-803 cells after transfection with hsa_circ_0000751 or control vector plasmids were detected by qRT-PCR. The blank group contains untreated cells. (b) The effect of hsa_circ_0000751 on cell viability was determined via CCK-8 assays. (c-d) The multiplication of GC cells was detected by clone formation assay and EdU assay. (e) The effect of hsa_circ_0000751 on cell invasion was determined via Transwell assay. (f) The effect of hsa_circ_0000751 on cell migration was determined via wound scratch assay. (g) TUNEL assay confirmed the incidence of apoptosis (Scale bar = 200 μm). (h) Apoptosis-related molecules expression was determined by western blot. Data are the means ± SD of triplicate determinants. *P < 0.05, **P < 0.01

To evaluate the effect of hsa_circ_0000751 in GC cells, we exogenously incorporated hsa_circ_0000751 plasmid or relative negative control into MKN-45 and MGC-803 cells. As depicted in Figure 2(a), hsa_circ_0000751 incorporation significantly elevated hsa_circ_0000751 expression in these cells. Notably, NUFIP2 transcript levels were unaffected (Figure 2(a)). Next, we performed functional assays to delineate the role of hsa_circ_0000751 on GC cell viability and proliferation rate. Using the CCK-8 assay, colony-formation assays and EdU assay, we showed that high expression levels of hsa_circ_0000751 resulted in a marked suppression of proliferation ability in both cell lines (Figure 2(b–d)). We further examined cell migration using transwell assays. As illustrated in Figure 2(e), hsa_circ_0000751 overexpression significantly reduced the invasive capacity of GC cells. Additionally, using wound healing assays, we showed that migration was strongly suppressed in hsa_circ_0000751-overexpressed cells, as opposed to cells with the negative control (figure 2(f)). To assess the effect of hsa_circ_0000751 on apoptosis, we performed TUNEL assay and western blotting. Cells with high expression levels of hsa_circ_0000751 exhibited more apoptosis than the cells with the negative control (Figure 2(g)). Moreover, apoptotic proteins such as cleaved caspase-3, Bak, and Bax proteins were markedly elevated upon hsa_circ_0000751 overexpression relative to the cells with the negative control. Conversely, Bcl-2 protein was downregulated under the same conditions (Figure 2(h)). Based on these results, it was inferred that hsa_circ_0000751-overexpressing cells induced apoptosis in GC cells.

### Hsa_circ_0000751 suppresses the growth of GC tumors in vivo

**Figure 3.**
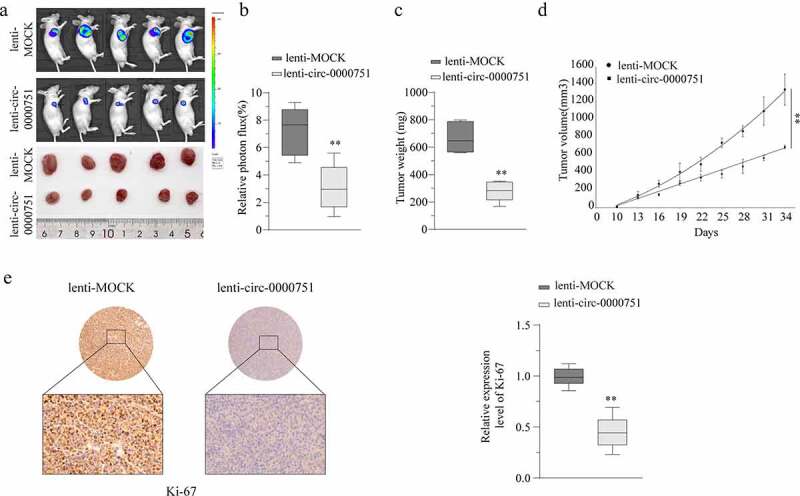
Hsa_circ_0000751 suppresses the growth of GC tumor in vivo. (a-d) MKN-45 cells were transfected with mock or lentivirus-hsa_circ_0000751-overexpression vectors. The stable hsa_circ_0000751 overexpression MKN-45 cells (5 × 10^6^ cells) were injected into nude mice, and tumors were allowed to develop for 1, 3 and 5 weeks. Representative images of the mice are shown, and the tumor weight and volumes were determined. (e) Ki-67 staining by immunohistochemical analysis of the xenograft tumors. Data are the means ± SD of triplicate determinants. **P < 0.01

To establish the role of hsa_circ_0000751 *in vivo*, we subcutaneously administered MKN-45 cells containing either hsa_circ_0000751 plasmid or negative control into the forelimb axilla of nude mice (n = 5) and monitored the development of tumors by observing luciferase intensities on a fortnightly basis. By week five, the mice carrying hsa_circ_0000751-overexpressed cells exhibited substantially small tumors, as evidenced by their growth rates, weights, and volumes, compared to mice carrying cells with the negative control (Figure 3(a–d)). Next, we harvested the tumor for Ki-67 staining, which revealed that hsa_circ_0000751-overexpressed mice had weak staining and low proliferation rate as compared to mice carrying cells with negative control (Figure 3(e)). Taken together, these data suggest that hsa_circ_0000751 overexpression can effectively suppress GC tumorigenesis *in vivo*.

### Hsa_circ_0000751 serves as an miR-488 sponge

**Figure 4.**
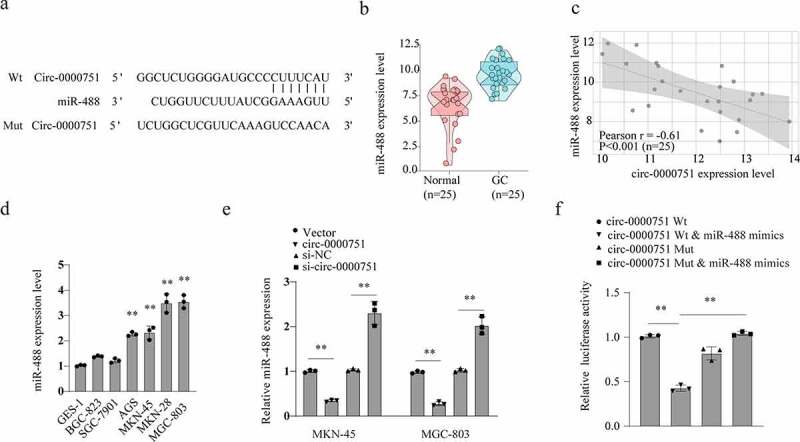
Hsa_circ_0000751 acted as a molecular sponge for miR-488. (a) Schematic representation of the targeting sites between hsa_circ_0000751 and the miR-488. (b) The Pearson’s correlation coefficients were used to evaluate the correlation between hsa_circ_0000751 and miR-488 in GC tissues (n = 25) (r = −0.61, P < 0.001). (c) The relative expression of miR-488 in 25 GC tissues was significantly higher than that in adjacent tissues. (d) qRT-PCR analysis of miR-488 expression in GES-1 and GC cell lines. (e) The effects of hsa_circ_0000751 on the expression of miR-488 were detected by qRT-PCR. (f) The luciferase activity of wild type hsa_circ_0000751 or mutant hsa_circ_0000751 after transfection with miR-488 mimic or inhibitor in HEK-293 T cells. Data are the means ± SD of triplicate determinants. **P < 0.01

To elucidate the underlying pathways involved in hsa_circ_0000751-mediated suppression of tumorigenesis, we scanned for potential targets of hsa_circ_0000751 using the miRNA target-prediction software (Arraystar made, based on TargetScan and miRanda databases). We discovered that hsa_circ_0000751 shared sequence homology with miR-488 (Figure 4(a)). Next, we evaluated miR-488 expression in the 25 pairs of GC tissues and adjoining healthy tissues, and found that miR-488 expression was significantly higher in GC tissues than in healthy tissues (Figure 4(b)). Moreover, there appeared to be a marked inverse relationship between miR-488 and hsa_circ_0000751, as evidenced in the 25 GC samples (r = −0.61, p < 0.001), as opposed to controls (Figure 4(c)). To test this further, we examined miR-488 levels in GES-1 and six different GC cell lines. Based on our results, the miR-488 transcript was found to be abundant in GC cells (Figure 4(d)). Additionally, we tested the relationship between hsa_circ_0000751 and miR-488 using qRT-PCR analysis. We demonstrated that high expression levels of hsa_circ_0000751 resulted in low levels of miR-488 in MKN-45 and MGC-803 cells, whereas hsa_circ_0000751 deficiency, caused by target-specific siRNA, upregulated the expression of miR-488 (Figure 4(e)). We further tested this inverse relationship using a luciferase reporter assay. Exogenous incorporation of miR-488 mimics strongly suppressed the luciferase activity of WT hsa_circ_0000751, which had an appropriate binding site for miR-488, but failed to suppress the activity of MUT hsa_circ_0000751, which carried a defective binding site for miR-488 (figure 4(f)). Collectively, these data indicate that hsa_circ_0000751 interacts directly with miR-488 to inhibit its action. In other words, hsa_circ_0000751 serves as an miR-488 sponge.

### Hsa_circ_0000751 inhibits GC cell proliferation and invasion by targeting miR-488

**Figure 5.**
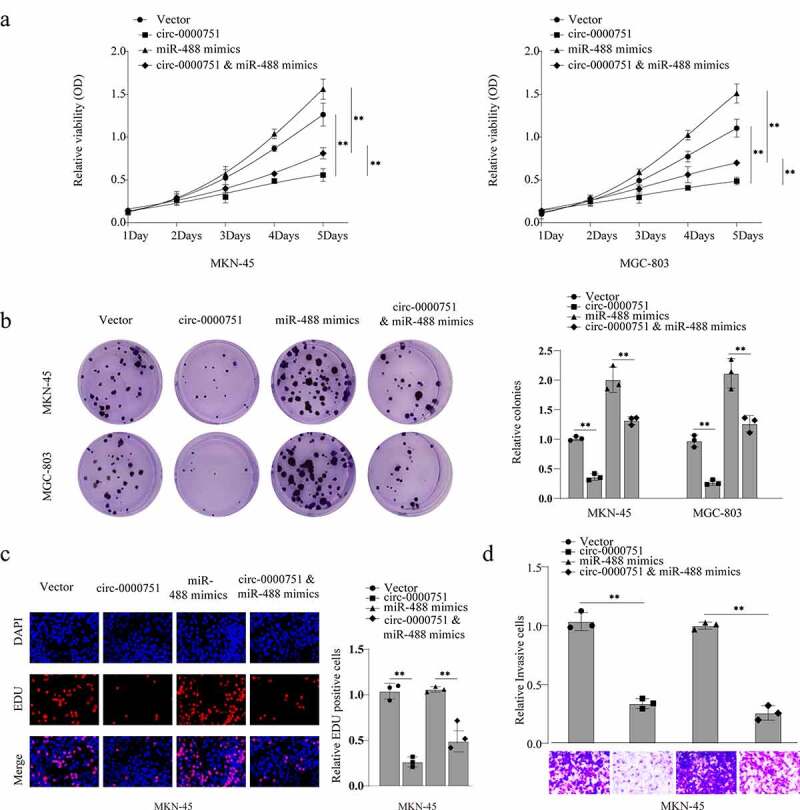
Hsa_circ_0000751 inhibits GC cell proliferation and invasion by targeting miR-488. MKN-45 and MGC-803 cells transfected with control vector, miR-488, hsa_circ_0000751, or miR-488 + hsa_circ_0000751. Then the ability of cell cloning, proliferation and invasion was, respectively, assessed by (a) CCK-8 assay, (b) colony formation assay and (c) transwell invasion assay. Data are the means ± SD of triplicate determinants. **P < 0.01

Given that hsa_circ_0000751 reduces GC cell growth and invasion, we next explored whether the cellular activities of hsa_circ_0000751 are mediated through the sponging of miR-488. Therefore, we conducted rescue experiments via co-transfection of MKN-45 and MGC-803 cells with miR-488 mimics and hsa_circ_0000751 expression vectors. Using CCK-8, colony-formation, and EdU assays, we demonstrated that GC cells incorporated with both hsa_circ_0000751 plasmids and miR-488 mimics exhibited higher cell proliferation than cells transfected with hsa_circ_0000751 plasmids alone (Figure 5(a–c)). This suggests a role of miR-488 mimics in reversing the hsa_circ_0000751-induced GC growth. In addition, we also demonstrated that hsa_circ_0000751 suppressed MKN-45 cell invasion, which was also attenuated in the presence of miR-488 (Figure 5(d)).

### Hsa_circ_0000751 modulates UQCRC2 level by sponging miR-488

**Figure 6.**
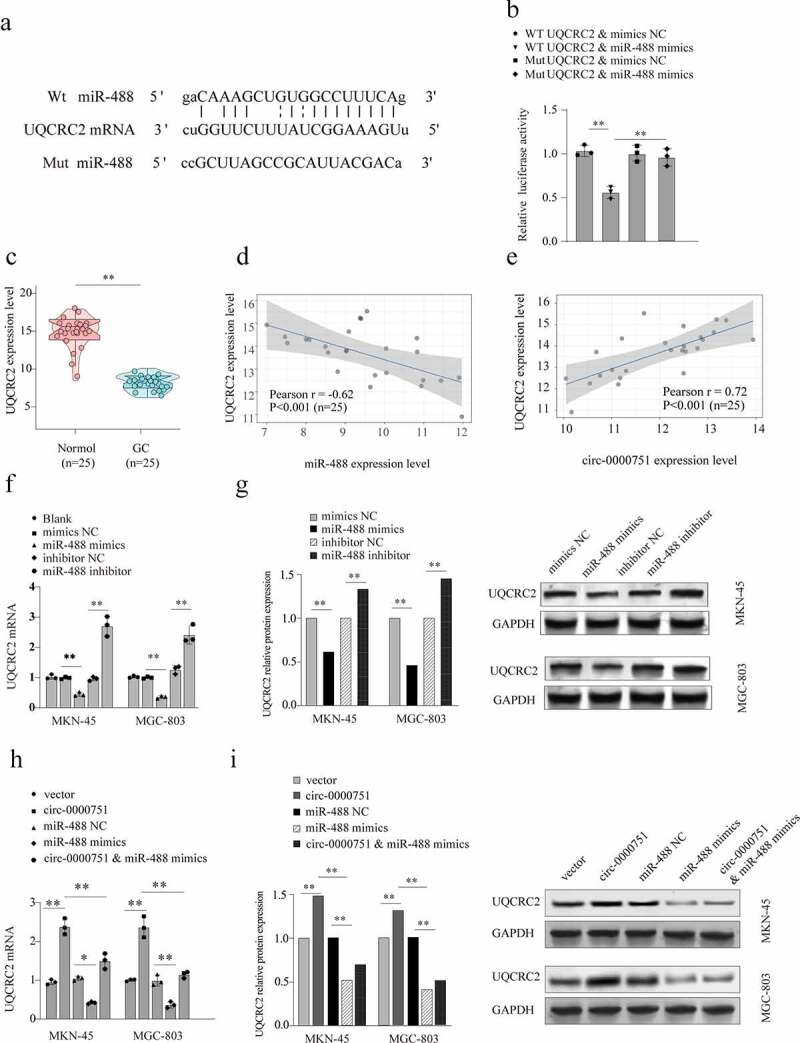
Hsa_circ_0000751 regulates the expression of UQCRC2 by sponging miR-488. (a) Schematic representation of the targeting sites between miR-488 and UQCRC2, which was predicted by Targetscan. (b) The luciferase reporter vector carrying wild type of UQCRC2 or mutant type of UQCRC2 was co-transfected with miR-488 mimics or mimic-NC. (c) UQCRC2 mRNA levels in 25 GC tissue specimens and matched non-carcinoma tissue specimens. (d) The Pearson’s correlation coefficients were used to evaluate the correlation between miR-488 and UQCRC2 in GC tissues (n = 25) (r = −0.62, p < 0.001). (e) The correlation analysis between the RNA level of hsa_circ_0000751 and UQCRC2 in 25 GC tissues (r = 0.72, p < 0.001). (f-g) The mRNA expression level and protein level of UQCRC2 in MKN-45 and MGC-803 cells after transfection with miR-488 mimics alone, miR-488 inhibitors or the corresponding negative controls. The blank group contains untreated cells. (h-i) Hsa_circ_0000751 could significantly promote the mRNA and protein expression of UQCRC2, and the promotion was retarded after co-transfecting with miR-488 mimics. Data are the means ± SD of triplicate determinants. **P < 0.01

To identify genes targeted by hsa_circ_0000751 via its negative regulation of miR-488, we used TargetScan to predict potential targets of miR-488. Based on our analysis, UQCRC2 was predicted to bind to miR-488 with a high affinity score (Figure 6(a)). Additionally, we verified our results using a dual-luciferase reporter assay. miR-488 overexpression strongly suppressed the luciferase response of WT UQCRC2, but not MUT UQCRC2, in 293 T cells (Figure 6(b)). Taken together, these results validate direct binding of UQCRC2 to miR-488. We then verified the expression of UQCRC2 in 25 GC samples and their corresponding healthy controls. We found that UQCRC2 expression was substantially downregulated in GC tissues (Figure 6(c)). Subsequent Pearson correlation analysis demonstrated a strong inverse association between the levels of miR-488 and UQCRC2 (r = −0.62, P < 0.001) (Figure 6(d)). Furthermore, we conducted a Pearson correlation analysis, which demonstrated a positive association between hsa_circ_0000751 and UQCRC2 levels in 25 GC tissues (r = 0.72, p < 0.001), as opposed to matched healthy tissues (Figure 6(e)). Then, incorporation of miR-488 mimics into MKN-45 and MGC-803 cells significantly lowered UQCRC2 levels, whereas miR-488 inhibitors markedly augmented UQCRC2 levels (figure 6(f)). Similarly, the protein levels of UQCRC2 were substantially low in GC cells (Figure 6(g)). Next, to elucidate the underlying mechanism by which hsa_circ_0000751 modulates UQCRC2 expression in GC cells, we overexpressed hsa_circ_0000751 in MKN-45 and MGC-803 cells and quantified UQCRC2 transcript levels using qRT-PCR. As shown in Figure 6(h), hsa_circ_0000751 overexpression significantly increased UQCRC2 transcript levels. Furthermore, this increase in UQCRC2 levels was attenuated by overexpression of miR-488 mimics. Similarly, we also demonstrated that high expression levels of hsa_circ_0000751 upregulated UQCRC2 protein expression, whereas miR-488 mimics, in the presence of hsa_circ_0000751, reduced UQCRC2 protein levels in MKN-45 and MGC-803 cells (Figure 6(i)). In summary, these data indicate that hsa_circ_0000751 positively regulates UQCRC2 expression by sponging miR-488.

### Hsa_circ_0000751 suppresses proliferation and invasion through regulation of the miR-488/UQCRC2 axis in GC cells

**Figure 7.**
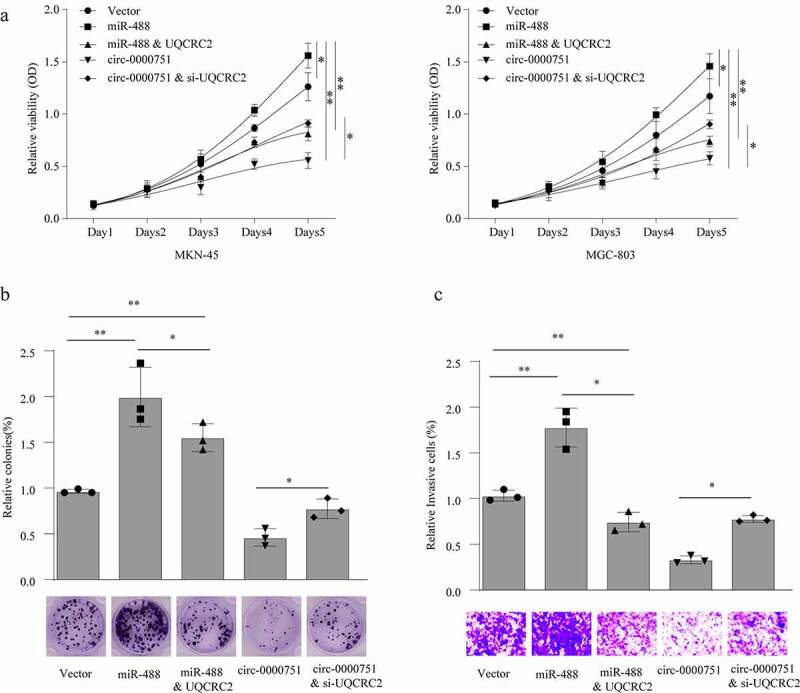
Hsa_circ_0000751 suppresses proliferation and invasion through modulation of the miR-488/UQCRC2 axis in GC cells. MKN-45 and MGC-803 cells transfected with control vector, miR-488 mimics, hsa_circ_0000751 overexpression plasmids, UQCRC2 plasmids or si-UQCRC2. cloning, proliferation and invasion was, respectively, assessed by (a) CCK-8 assay, (b) colony formation assay and (c) transwell invasion assay. Data are the means ± SD of triplicate determinants. *P < 0.05, **P < 0.01

Next, we investigated whether the hsa_circ_0000751-mediated regulation of GC cell proliferation and invasion occurs via the miR-488/UQCRC2 axis. For this, we employed CCK-8, colony-formation, and invasion assays, which showed that miR-488–overexpressing MKN-45 and MGC-803 cells exhibited substantially higher viability than cells with negative controls. Additionally, cells incorporated with both the miR-488 mimic and UQCRC2 had low viability (Figure 7(a–b)). We also showed that UQCRC2 knockdown severely weakened hsa_circ_0000751-mediated suppression of proliferation. In addition, we demonstrated that MKN-45 invasion ability increased with miR-488 overexpression, but was impaired in the presence of UQCRC2 plasmids. Conversely, UQCRC2 insufficiency increased hsa_circ_0000751-mediated cell invasion (Figure 7(c)). Taken together, these results demonstrate that hsa_circ_0000751 suppresses GC cell growth and invasion via its effect on the miR-488/UQCRC2 axis.

## Discussion

CircRNAs are a group of circular ncRNAs that possess no 5ʹ–3ʹ polarity or polyA tails [23]. They have been shown to have great potential as cancer biomarkers, as analyzed via expression profiling [24–26]. For example, Gu et al. [27] distinguished 440 differentially expressed circRNAs using microarray analysis of six matched GC tumors and adjacent healthy tissues. In another study, circFAT1(e2) was found to suppress tumors in GC cells via cytoplasmic modulation of the miR-548 g/RUNX1 axis and the nuclear regulation of YBX1 [28]. Likewise, Zhang et al. [29] explored sequencing data from the Cancer Genome Atlas to show that circLARP4 serves as an anti-oncogene in GC patients. Here, we employed the GEO database to identify differentially regulated circRNAs in six GC tissues and six adjacent tissues. Among the differentially regulated circRNAs, hsa_circ_0000751 expression level showed the largest reduction in GC tissues. Functionally, we revealed that hsa_circ_0000751 strongly suppressed the growth and invasion of GC cells. These data indicate that hsa_circ_0000751 acts as a tumor-suppressor gene in GC tissue samples and cells. It also raises the possibility of employing hsa_circ_0000751 as a new diagnostic biomarker and for therapy in patients with GC.

The competitive endogenous RNA (ceRNA) hypothesis states that miRNAs with sequence homology with target transcripts regulate the transcription of their target transcripts [30]. CircRNAs have been reported to have high-frequency binding sites for miRNAs, suggesting a possible role as miRNA sponges. This hypothesis unifies the roles of circRNA, pseudogene transcripts, long non-coding RNAs, and miRNAs [31]. Indeed, hsa_circ_0013958 in lung adenocarcinoma was shown to competitively interact with miR-134 and attenuate its repression of cyclin D1, thereby accelerating cell proliferation [32]. Furthermore, hsa_circ_0000117 was shown to accelerate tumor cell growth by sponging miR-337-3P [33]. Here, using miRNA-targeting prediction analysis, we demonstrated aberrant expression of miR-488, along with its targeted association with hsa_circ_0000751, in GC versus healthy tissues. Based on our results, miR-488 levels were negatively correlated with hsa_circ_0000751 in GC cell lines. Consistent with our findings, another study demonstrated that overexpression of miR-488 drove the progression of colorectal cancer via its regulation of plant homeo domain finger protein 8 (PHF8) [34]. Similarly, miR-488 expression was shown to be substantially high in NSCLC tissues and cells, which correlates with a clinically advanced stage of the disease and poor survival in patients [35]. Taken together, our results indicate that hsa_circ_0000751 possesses an anti-oncogenic role in GC that is imparted, in part, by sponging miR-488.

Based on the ceRNA hypothesis, circRNA can serve as a ceRNA in the modulation of miRNA target gene expression. Our TargetScan analysis projected UQCRC2 to have binding sites for miR-488. UQCRC2 encodes core protein 2, which is one of the 11 structural subunits of mitochondrial complex III. Multiple studies have shown that UQCRC2 plays a crucial role in the progression and metastasis of numerous cancers, including colorectal cancer [36], breast cancer [37], testicular cancer [38], among others. However, the function of UQCRC2 and specific miRNAs in GC progression remains unknown. In this study, we showed that miR-488 overexpression markedly suppressed UQCRC2 transcript and protein expression, whereas miR-488 deficiency resulted in elevated UQCRC2 levels. Using dual-luciferase reporter assays, we confirmed that miR-488 can directly target UQCRC2. In previous studies, we discovered that UQCRC2 expression remains substantially low in GC tissues and is correlated with a better prognosis in GC patients [14]. Here, we showed that UQCRC2 knockdown suppressed the hsa_circ_0000751-mediated action on the proliferation and invasion of GC cells. These results strongly suggest the presence of a hsa_circ_0000751-miR-488-UQCRC2 axis in GC, whereby hsa_circ_0000751 sequesters the activity of miR-488 to promote the upregulation of UQCRC2 and regression of GC.

## Conclusion

In summary, we discovered the miR-488 sponging activity of hsa_circ_0000751, which upregulates UQCRC2 expression and suppresses the proliferation and invasion of GC cells. This study provides insight into the novel hsa_circ_0000751-miR-488-UQCRC2 axis involved in GC progression. However, there are certain limitations of this research. The potential signaling pathways and molecular mechanisms related to circRNAs in the regulation of GC cell proliferation remain to be investigated. Future in-depth clinical investigations involving a larger sample size are both urgent and crucial to the advancement of GC early detection and therapy.

## Data Availability

All data generated or analyzed during this study are included in this published article.
